# Detecting poststroke epilepsy in nationwide administrative data: A validation study using Swedish registers

**DOI:** 10.1371/journal.pone.0329012

**Published:** 2025-08-12

**Authors:** André Idegård, David Larsson

**Affiliations:** 1 Department of Clinical Neuroscience, Institute of Neuroscience and Physiology, Sahlgrenska Academy, Gothenburg University, Gothenburg, Sweden; 2 Wallenberg Center of Molecular and Translational Medicine, Gothenburg University, Gothenburg, Sweden; 3 Department of Neurology, Sahlgrenska University Hospital, Member of the ERN EpiCARE, Gothenburg, Sweden; National Cerebral and Cardiovascular Center: Kokuritsu Junkankibyo Kenkyu Center, JAPAN

## Abstract

**Objective:**

Healthcare administrative data often rely on the International Classification of Diseases (ICD) system, which lacks specific codes to identify etiological subgroups of epilepsy. Combining indicators for epilepsy and potential etiologies is possible, but such approaches require validation. This study aimed to validate methods for identifying poststroke epilepsy (PSE) in Swedish administrative data.

**Methods:**

The algorithms were based on combinations of ICD-10 codes for stroke and seizures, with some also incorporating antiseizure medication prescriptions. We focused on positive predictive values (PPVs), using medical records as the reference standard. We identified individuals in the National Patient Register with a primary inpatient diagnostic code for stroke (I61 or I63) during 2005–2010 and a first-ever seizure-related code (G40, G41, or R56.8), occurring more than seven days post-stroke. To facilitate access to medical records, only patients who were deceased at data extraction (Jan 16, 2021) were eligible. A nationwide random sample of 500 patients was selected, with the intended sample for medical record review being 250. Medical records were reviewed before processing the administrative data.

**Results:**

Records were obtained for 321 patients (median age 78; 56% males), with no significant differences in characteristics between those included and the rest of the sample. Across different algorithms, PPVs ranged from 84.1% (95% CI: 79.2–88.3) to 92.5% (95% CI: 87.3–96.1). Relative coverage ranged from 60% to 89% compared to the most inclusive algorithm.

**Significance:**

Our findings demonstrate the potential of administrative data to reliably identify PSE cases, supporting the use of these algorithms for large-scale studies of treatment and outcomes. Stricter algorithms, limited to G40 codes for epilepsy or requiring ASM prescriptions, improve accuracy but at the cost of missing more cases. Limitations include the inability to calculate sensitivity due to study design, and the need for local validation before use in other healthcare systems.

## Introduction

Healthcare administrative data—generated during everyday clinical practice for non-research purposes—are increasingly being used for epilepsy research. In Sweden and neighboring Scandinavian countries, such data is stored in national registers managed by government authorities, providing researchers with the opportunity to collect population-wide samples and track them over time. However, the reliability of research based on administrative data depends on the accuracy of the algorithms used to identify cases. Without proper validation, there is a risk of misclassification, leading to effect measures being biased towards null values [1].

Poststroke epilepsy (PSE) is an area that could benefit from the use of administrative data. Its patient population, risk factors, and treatment considerations differ substantially from other epilepsy etiologies. Cerebrovascular disease is the most common cause of new-onset epilepsy after middle age and accounts for approximately 14–21% of all incident epilepsy cases in Europe [2]. The long-term cumulative incidence of PSE varies by study design, but has been estimated at approximately 6.4% in the largest prospective population-based study [3]. Seizures are generally about twice as common after intracerebral hemorrhage compared to acute ischemic stroke [4,5]. Administrative data is particularly valuable for studying patients who are difficult to recruit or follow over time, such as older adults with multiple comorbidities. With an aging population and increasing stroke survival, PSE is likely to become an even more important area of care [2].

While numerous epilepsy case identification algorithms have been validated, none have been formally validated in Sweden, where our work is based, nor have they focused on accurately distinguishing specific etiological subgroups [6]. To date, only one study, conducted in the United States using Medicare claims data, has specifically evaluated an algorithm to identify PSE [7]. That algorithm relied on electroencephalography data not available in Swedish registers, limiting its generalizability. As such, this study aimed to validate algorithms to identify and monitor individuals with PSE in Sweden, leveraging national registers that cover the entire population.

## Materials & methods

### Study design and setting

The primary aim of this retrospective cohort study was to validate the accuracy of our register-based algorithms in identifying patients with PSE, using medical records as the reference standard measure.

### Definition of PSE candidate algorithms

The structure of our candidate algorithms was informed by previous validation studies of epilepsy in administrative data (not specific to PSE), which have shown that ICD codes for epilepsy (ICD-10: G40) yield moderate-to-high PPVs, particularly when combined with ASM prescriptions [6]. Seizure symptom codes (ICD-10: R56.8) can identify additional cases, but are generally less specific. Information on electroencephalography is not available in Swedish administrative data.

In the context of PSE, all algorithms required a prior stroke (ICD-10: I61 or I63 as the primary diagnosis during inpatient care), no seizure-related ICD codes prior to the stroke (ICD-10: G40, G41, R56.8), and a seizure-related code occurring more than 7 days after the stroke hospitalization. The 7-day time window was to reduce the risk of capturing isolated acute symptomatic seizures, which are not considered epilepsy by current definitions. To ensure maximum case capture, we started with the broadest possible algorithm, a single ICD code for stroke and a single ICD code for seizures, including the symptom code R56.8. We then gradually made the algorithm criteria stricter by requiring, in different combinations, an ICD code for epilepsy (ICD-10: G40), multiple seizure-related codes, and prescriptions of ASMs (ATC code N03*).

In total, we utilized three Swedish national registers with complete coverage during the study period: The National Patient Register [8], which records dates and diagnostic codes for inpatient and specialized outpatient care; the National Prescribed Drug Register [9], which encompasses prescription details on all drugs dispensed at pharmacies; and the Cause of Death register [10], which includes mortality data.

### Study population

**Fig 1:**
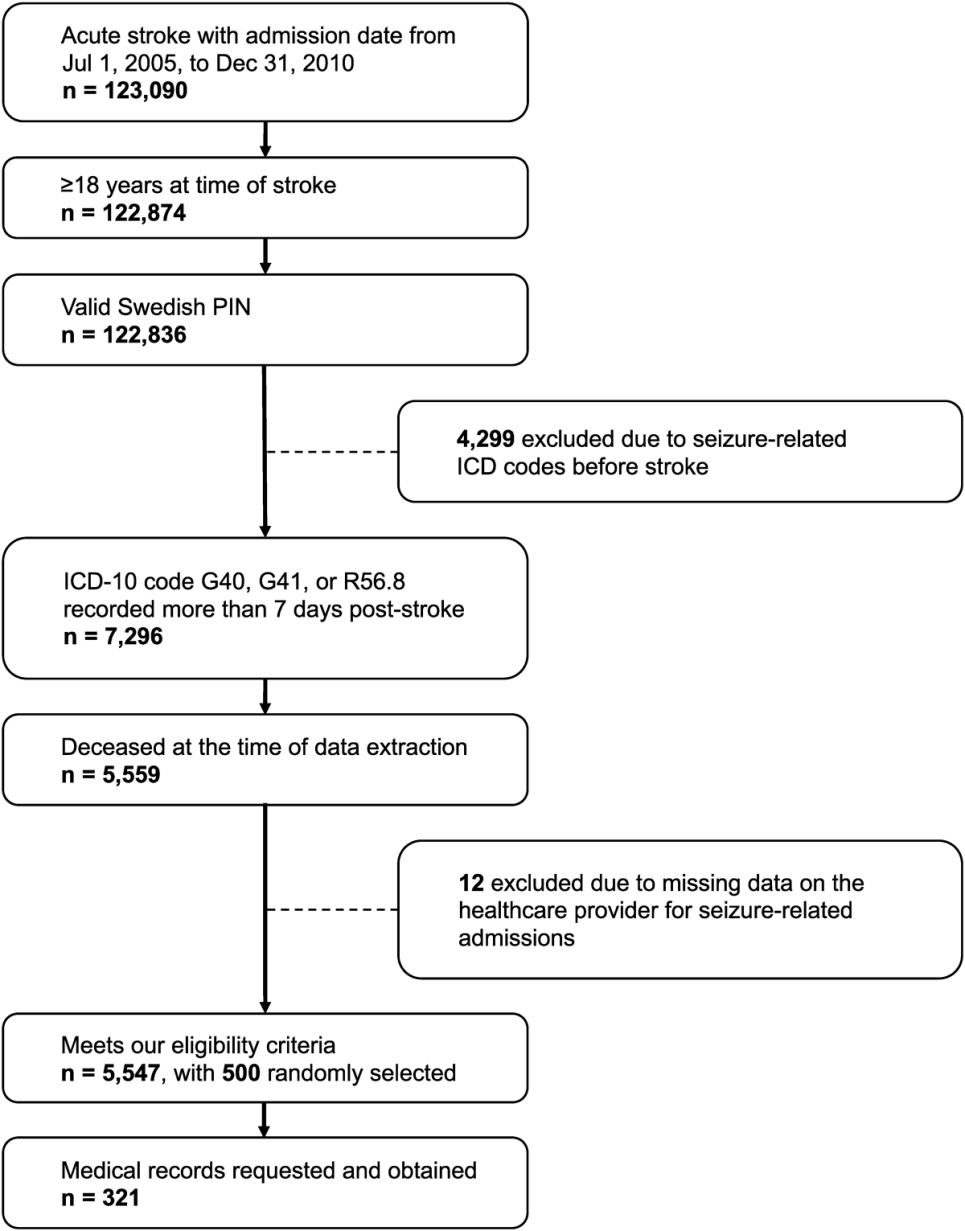
Flow chart of study eligibility criteria. The cohort was population-based and drawn from the Swedish National Patient Register.

The eligibility process is illustrated in detail in Fig 1. In short, the Swedish National Board of Health and Welfare identified patients with a diagnostic code for stroke during inpatient care (ICD-10: I61or I63 as the primary diagnosis), followed by a first-ever diagnostic code for seizures (R56.8, G40, or G41 in any diagnosis field) with at least one occurrence more than seven days post-stroke. Given Sweden’s strict laws on accessing and sharing medical data, we only included patients who were deceased at the time of data extraction. We added this criterion to minimize privacy risks and enable healthcare providers to more easily comply with our request for medical records. Due to the increased mortality after stroke, we anticipated that the vast majority of patients would have passed away before the point of data extraction.

### Data collection and assessment of medical records

Based on the eligibility criteria, the Swedish National Board of Health and Welfare provided us with a nationwide random sample of 500 individuals. Accessing personal data on the whole eligible cohort would not have been justifiable from a data integrity perspective. The register data extraction took place on January 16, 2021. We received information on individual identifiers, dates, and healthcare providers for all specialized healthcare encounters with registered diagnostic codes related to stroke (ICD-10: I61, I63, I64) or seizures (R56.8, G40, G41), including inpatient care, doctor’s appointments, and emergency room visits. We contacted the relevant healthcare providers and asked them to supply the corresponding medical records as the reference standard. Swedish healthcare is decentralized, using different electronic health records systems across administrative regions, so we had to request paper copies via mail. Each healthcare provider was compensated for their time. Medical records were ordered and received in batches until April 27, 2022.

We did not, however, obtain medical records for the entire sample. In the planning stage, we decided it would be within the study’s scope to examine about 250 records. We estimated that this would give 95% confidence intervals (95% CI) with a margin of error of around ±5%, which we considered acceptable. We anticipated that some healthcare providers would decline our requests due to uncertainties about privacy laws or resource constraints (such as limited staff). As a result, we initially targeted information on 500 individuals, even though our intended sample for the medical record review was roughly 250. Ultimately, most healthcare providers that responded complied with our request; consequently, we terminated further requests after obtaining records on 250 individuals. All subsequently received records were examined (total n = 321).

A junior physician (AI) reviewed the medical records in October and November 2023. PSE was defined according to the current ILAE criteria, i.e., at least one remote symptomatic (‘late’) seizure attributable to a stroke [11]. Seizures occurring within seven days of a stroke were classified as acute symptomatic. In the majority of cases, the documentation regarding seizure assessment and etiology was unambiguous. A neurologist subspecializing in epilepsy (DL) validated a sample of the cohort (initial joint review) and secondarily reviewed all records flagged as posing interpretive challenges (totaling 65 records [20%]). It was predetermined that the neurologist would review all cases with a history of multiple brain disorders (‘competing’ etiologies) or a time gap exceeding three years between stroke and seizure onset (PSE typically develops within the first few years). Both reference standard readers were blinded for the results of the register-based algorithms. However, since the ICD codes in the National Patient Register have originally been entered by physicians during each patient encounter, they are also part of the medical records.

### Validation methods and statistical analysis

The medical record review divided patients into two groups: ‘PSE confirmed’ (true positives) and ‘absence of PSE confirmed’ (false positives). The study’s primary measure is the PPV, calculated as the proportion of true positives among all positives identified by each register-based algorithm. To calculate 95% CIs, we used the Clopper-Pearson method for binomial proportions. We did not conduct any statistical comparisons between algorithms. Cases that could not be confirmed or ruled out as having PSE were classified based on the underlying reason and then excluded from the main analysis. For cases deemed unassessable due to incomplete medical records, we analysed G40 (epilepsy) registrations and ASM prescriptions to get additional insights into the likelihood of PSE. Fisher’s exact test and the Mann–Whitney U-test were used for statistical evaluation of group differences, with a significance level of 0.05. As a sensitivity analysis, we also report results from a “worst-case scenario,” where all patients with incomplete records were assumed not to have PSE.

### Ethical considerations

Ethical approval was not required for this study under Swedish law, as it only includes deceased individuals. The Swedish Ethical Review Authority issued an advisory opinion, stating they saw no ethical concerns with the project. As the study does not involve living participants, informed consent was not applicable.

Data were not pseudonymized before access; full individual identifiers were available to us, as they were necessary to order the corresponding medical records. The data was handled in accordance with Swedish regulations.

## Results

The cohort comprised 321 individuals (median age 78 [Q1-Q3: 70–84]; 56% males). Table 1 displays the demographics and clinical characteristics of the cohort. There were no significant differences in characteristics between individuals included in the cohort and those whose records were not ordered or received (S1 Table).

**Table 1:** Characteristics of the cohort as extracted from the national registers, stratified according to poststroke epilepsy (PSE) status as verified through medical records. *P* = p-value.

	All n = 321	PSE confirmed n = 228	Absence of PSE confirmed n = 43	*P*
Age at first seizure-related diagnostic code, years (median, IQR)	78 (70-84)	78 (72-84)	75 (66-85)	.186
Sex	n (%)			1.00
Male	181 (56)	127 (56)	24 (56)	
Female	140 (44)	101 (44)	19 (44)	
Index stroke type				.236
Acute ischemic stroke	274 (85)	196 (86)	34 (79)	
Intracerebral hemorrhage	47 (15)	32 (14)	9 (21)	
Diagnostic code for seizures (G40, G41, or R56.8)				
Primary diagnosis	259 (81)	202 (89)	21 (49)	<.001
Any diagnosis field	321 (100)	228 (100)	43 (100)	1.00
Multiple occurrences	210 (65)	155 (68)	27 (63)	.596
Diagnostic code for epilepsy (G40)				
Primary diagnosis	182 (57)	148 (65)	15 (35)	<.001
Any diagnosis field	245 (76)	184 (81)	30 (70)	.151
Multiple occurrences	183 (57)	136 (60)	24 (56)	.736
Time between stroke and first seizure-related diagnostic code				
Median number of days (IQR)	322 (141-700)	329 (153-631)	114 (0-641)	<.001
0–7 days	30 (9)	14 (6)	15 (35)	
≤ 1 years	173 (54)	124 (54)	30 (70)	
≤ 2 years	242 (75)	180 (79)	34 (79)	
Dispensed ASM^†^				
Anytime	258 (80)	194 (84)	32 (74)	.115
First dispensation after stroke	239 (75)	191 (84)	19 (44)	<.001
First dispensation after poststroke seizure	214 (67)	174 (76)	15 (35)	<.001

^†^Pregabalin and gabapentin were not included, as they are frequently used for other indications, for example, central post-stroke pain.

### Medical record review

We received medical records from 27 healthcare entities, including both individual hospitals and administrative regions responsible for multiple hospitals. The review confirmed epileptic seizures in 291 patients (91% of the cohort), with 264 patients having confirmed unprovoked seizures. The outcomes of the medical record review are displayed in Table 2. In 36 cases (11% of the cohort), the medical records we received were incomplete to such an extent that no reasonable conclusions could be drawn. About half of these cases (n = 16) came from the same three healthcare providers (which contributed a total of 42 cases).

**Table 2:** Outcomes of medical record review for validation of poststroke epilepsy (PSE) status.

	All n = 321
**PSE confirmed, n (%)**	228 (71)
**Absence of PSE confirmed, n (%)**	43 (13)
No stroke diagnosis (erroneous coding)	7 (2)
No seizure diagnosis (erroneous coding)	1 (0)
Unprovoked seizure or epilepsy before stroke	13 (4)
Acute symptomatic seizures only	17 (5)
Other epilepsy etiology	5 (2)
**Unresolved cases, n (%)**	14 (4)
Unclear if unprovoked	3 (1)
Unclear etiology due to co-existing brain disorders	7 (2)
Unclear etiology due to other reason	3 (1)
Due to insufficient documentation	1 (0)
**Incomplete medical records, n (%)**	36 (11)

### Assessment of register-based algorithms

The broadest algorithm, i.e., the one corresponding to our eligibility criteria, yielded a PPV of 84.1% (95% CI: 79.8–88.5). For that algorithm, we also provide a chart of the PPV stratified by survival after the first seizure code (S1 Fig). Table 3 displays the PPVs and the relative coverage (compared to the broadest algorithm) of our register-based algorithms. The PPV of the broadest algorithm was higher, but not statistically significant, for acute ischemic stroke (85.2%) compared to ICH (78.0%).

**Table 3:** Positive predictive values (PPVs) of the register-based algorithms to identify individuals with poststroke epilepsy (PSE). All algorithms required (i) a diagnostic code for stroke as primary diagnosis during inpatient care, (ii) at least one occurrence of a disease or symptom code for seizures (ICD-10: G40, G41 or R56.8) more than seven days after stroke admission, and (iii) no registrations of seizure-related diagnostic codes prior to the stroke.

	PSE, n	Not PSE, n	PPV, % (95% CI)	Relative coverage^§^, %
Stroke, + diagnostic code for seizures^†^	228	43	84.1 (79.2-88.3)	100
Stroke, + multiple occurrences of diagnostic codes for seizures^†^	155	27	85.2 (79.2-90.0)	68
Stroke, + diagnostic code for seizures as primary diagnosis	202	21	90.6 (86.0-94.1)	89
Stroke, + diagnostic code for seizures^†^, + first-ever ASM^‡^ prescription after poststroke seizure	174	15	92.1 (87.2-95.5)	76
Stroke, + diagnostic code for epilepsy^†^	184	30	86.0 (80.6-90.3)	81
Stroke, + multiple occurrences of diagnostic codes for epilepsy^†^	136	24	85.0 (78.5-90.1)	60
Stroke, + diagnostic code for epilepsy as primary diagnosis	148	15	90.8 (85.3-94.8)	65
Stroke, + diagnostic code for epilepsy^†^, + first-ever ASM^‡^ prescription after poststroke seizure	149	12	92.5 (87.3-96.1)	65

^†^In any diagnosis field, meaning not limited to the primary diagnosis.

^‡^Pregabalin and gabapentin were not included, as they are frequently used for other indications, for example, central post-stroke pain.

^§^Relative coverage was calculated using the broadest algorithm as reference (set to 100%), representing the maximum number of PSE cases identifiable using register-based diagnostic coding.

Based on the observations in Table 1, a proportion of PSE patients had an ASM dispensed before their first seizure code. We, therefore, conducted an additional exploratory analysis (not pre-defined) requiring a first-ever ASM dispensation after stroke, and a G40 code for epilepsy. This algorithm yielded a PPV of 91.2% (95% CI: 86.0–94.9) and identified more PSE cases (n = 165) than when requiring the ASM dispensation to occur at or after the first seizure code.

### Sensitivity analysis of cases with incomplete records

Among patients with incomplete medical records, many had a G40 code for epilepsy, with a proportion similar to the rest of the cohort (72% vs. 70%; p = 0.85). The frequency of a first-ever ASM prescription after stroke was slightly lower, but not significantly different (67% vs. 75%; p = 0.31). In a “worst-case scenario” analysis, where we assumed that none of the 36 patients with incomplete records had PSE, the PPVs of the algorithms ranged between 74.3% and 86.6%. The algorithm combining stroke and seizure codes (G40, G41, R56.8) with an ASM prescription achieved a PPV of 83.3% (95% CI: 77.5–88.0).

## Discussion

In this nationwide study, we validated several register-based algorithms against medical records, all of which accurately identified patients with PSE, achieving PPVs ranging from 84.1% to 92.5%. As expected, the algorithm requiring a single ICD code for stroke and a single ICD code for seizures, including the symptom code R56.8, had the lowest PPV. Adding an ASM prescription as a prerequisite increased the PPV to 92% but, at the same time, made us exclude 24% of true PSE cases, illustrating the trade-off between PPV and coverage. Focusing solely on individuals with a G40 code for epilepsy omitted 19% of true PSE cases, with minimal gain in PPV. However, most cases in our cohort were diagnosed before the 2014 ILAE epilepsy definition update, which allowed diagnosing PSE after a single remote symptomatic seizure [11]. Accordingly, the coverage of the G40 code among single seizure patients has likely improved since 2014, and for those with recurrent seizures, the code may now be registered earlier in the disease course.

In a subset of patients (11%), the medical records we received were so incomplete that no reasonable conclusions could be drawn from them. The ICD codes in the National Patient Register originate from healthcare encounters with corresponding medical records; therefore, the missing data do not reflect the absence of clinical documentation but rather challenges in retrieval of medical charts. Accordingly, we believe the missing data is unlikely to be related to the patient’s PSE status. The proportion with G40 codes and ASM dispensations was comparable to the rest of the cohort, implying that missingness is unlikely to have introduced substantial bias. Nevertheless, even when assuming that none of the 36 patients with incomplete records had PSE, the PPVs of the algorithms still ranged between 74.3% and 86.6%, which is generally considered moderately high to high in the context of register-based or claims-based validation studies [6].

Seizure classification within the ICD coding system does not account for etiology, meaning that the only way to identify etiological subgroups in such administrative data is by combining a seizure-related code with one or more codes reflecting a potential etiology. However, the combination of two ICD codes does not necessarily imply a causal relationship, and in the context of stroke, there is also the risk of a seizure-related code reflecting early poststroke seizures. The high accuracy across all our algorithms suggests that (i) other etiologies are uncommon in the presence of a strong risk factor such as stroke, and (ii) patients with only acute symptomatic seizures typically did not receive seizure-related codes beyond 7 days after stroke admission.

Studies aiming to achieve even higher PPVs could utilize national stroke registers to accurately identify stroke patients (likely improving PPVs by ~2%), adopt stricter criteria to exclude patients with other ‘competing’ etiologies (e.g., brain tumors or dementia), or incorporate additional time-based rules, such as excluding patients with seizure codes or ASM dispensations occurring immediately after the stroke. However, our results suggest that algorithms incorporating stricter criteria or additional rules might impact coverage and generalizability more significantly than they improve accuracy, meaning that you might underestimate rates of occurrence and risk a skewed representation of the clinical spectrum. In register-based research, this is typically a greater issue for epidemiological studies than for analytical studies, which usually prioritize high PPVs to avoid false positives and minimize misclassification bias.

This is the second study overall to validate algorithms for detecting PSE and the first formal validation study for epilepsy case identification in Sweden. Our PPVs are similar to, or higher than, in the aforementioned Medicare claims-based PSE study, which reported a PPV of 86.5% (wide 95% CI between 71.2% and 95.5%) and a sensitivity of 20.6% for their best algorithm [7]. Interestingly, in that study, claims indicative of epilepsy (as primary, secondary, or tertiary diagnosis) showed very low sensitivity (5.4%), explaining why they had to rely on other factors such as EEGs or prescribed ASMs. Regarding epilepsy of mixed etiologies, a Danish validation study, using a study design and national registers similar to ours, found that ICD codes for epilepsy had an accuracy of 81% (95% CI: 75–87) [12]. In Sweden, a SUDEP study using medical records to determine causes of death reported that 90% of reviewed individuals with a prior G40 code fulfilled epilepsy criteria, which is consistent with our findings [13].

During the planning stage, we made two major decisions to increase feasibility, though these also introduce certain limitations. Firstly, we used indicators of stroke and epilepsy to identify the cohort, rather than reviewing medical records unconditionally, which would have been required to calculate sensitivity and specificity. Our focus on PPV was based on the fact that, in Swedish register-based research, particularly when studying treatment outcomes or risk factors, internal validity is usually the primary concern. This is mainly due to the National Patient Register’s complete coverage of inpatient and specialized outpatient care, including stroke units, neurology departments, and emergency rooms, which minimizes the risk of missing cases in these settings. While PSE cases not assessed in specialized care will undoubtedly go undetected, it is relatively uncommon, and against Swedish guidelines, for epilepsy patients to undergo work-up, diagnosis, and management exclusively in primary care. As a form of validation, using our most inclusive algorithm, the long-term cumulative incidence of epilepsy after a first-ever stroke would be estimated to be around 5.9%, and a bit higher (~7% [14]) if you only use stroke survivors in the denominator. This aligns well with results from other studies using primary sources like medical records or interviews with long-term follow-up after stroke, indicating that the majority of true cases are captured [3,5].

Secondly, we focused only on patients who were deceased at the time of data extraction, as personal data is not protected as rigorously after death. We were concerned that healthcare providers might not comply with our requests if we asked for medical records of living individuals, as fulfilling such requests would require time and resources – particularly if questions arose regarding the legality of providing records under privacy laws, which might necessitate involving legal experts. In a general epilepsy population, this approach might introduce selection bias, but in a cohort of older adults with high background mortality primarily due to cardiovascular comorbidity [15], we believed this impact would be minimal. In practice, the majority of eligible patients (76%) had indeed passed away before data extraction, and the long-term survivors of our cohort had equal or even higher PPVs. Therefore, we cannot rule out that estimates may be slightly conservative for the entire eligible population.

Other limitations of our study include its retrospective design, as our use of historical records may not fully reflect current coding behavior. Coding variability between individual hospitals or health service regions is another concern, but we have tried to mitigate this by selecting patients randomly from all over Sweden. Additionally, all healthcare providers adhere to the same ICD coding guidelines issued by the National Board of Health and Welfare. We did not record any formal interrater agreement measures; the initial reviews were conducted jointly, and later assessments followed a standardized process with secondary review in cases of uncertainty. Finally, the findings are based on Swedish data sources and may not be generalizable to other healthcare systems with different coding practices or register structures. Our methods may be transferable to other countries with similar registers, particularly in the Nordic region, but local validation would be necessary to ensure reliability.

Our findings suggest several potential applications. First, the proposed PSE case definitions seem to be accurate and can be used to identify population-based samples. This is particularly promising given that individuals with PSE are typically difficult to include or follow prospectively over time. Second, as the differences in PPVs between algorithms are relatively small, the choice of algorithm can be tailored to the study’s purpose. For epidemiological studies that require high coverage, we recommend using the broadest algorithm, which includes the symptom code R56.8. For outcome studies, requiring an ASM prescription is appropriate. However, to avoid excluding too many true PSE cases, it is beneficial to include patients with the symptom code R56.8, particularly for the years prior to the ILAE epilepsy definition update in 2014 [11]. For patients with seizure onset after 2014, we suggest requiring a G40 code for epilepsy, as the coverage of that code has likely improved since the update.

## Conclusions

Our findings demonstrate that simple and easy-to-adapt algorithms, based on ICD coding, and some incorporating data on ASM prescriptions, reliably identify PSE in Swedish administrative data. Stricter algorithms, limited to G40 codes for epilepsy or requiring ASM prescriptions, improve accuracy but at the cost of missing more cases. These findings support the use of administrative data for large-scale research on PSE treatment, outcomes, and epidemiology. Although our algorithms are based on data elements that are available in many countries, local validation would be necessary to ensure reliability across different healthcare systems.

## Supporting information

S1 Fig Positive predictive value (PPV, %) by survival time following first seizure code. Bar chart showing the PPV of our broadest algorithm, stratified by patient survival time in years following the first seizure-related code.
(TIF)

S1 Table Comparison of characteristics between individuals included in the study and those whose records were not ordered or received. (PDF)
