# Supplementary figures and images for: Detecting poststroke epilepsy in nationwide administrative data: A validation study using Swedish registers
Source: PLoS One. 2025 Aug 12;20(8):e0329012. doi: 10.1371/journal.pone.0329012 (PMC12342257; doi:10.1371/journal.pone.0329012)

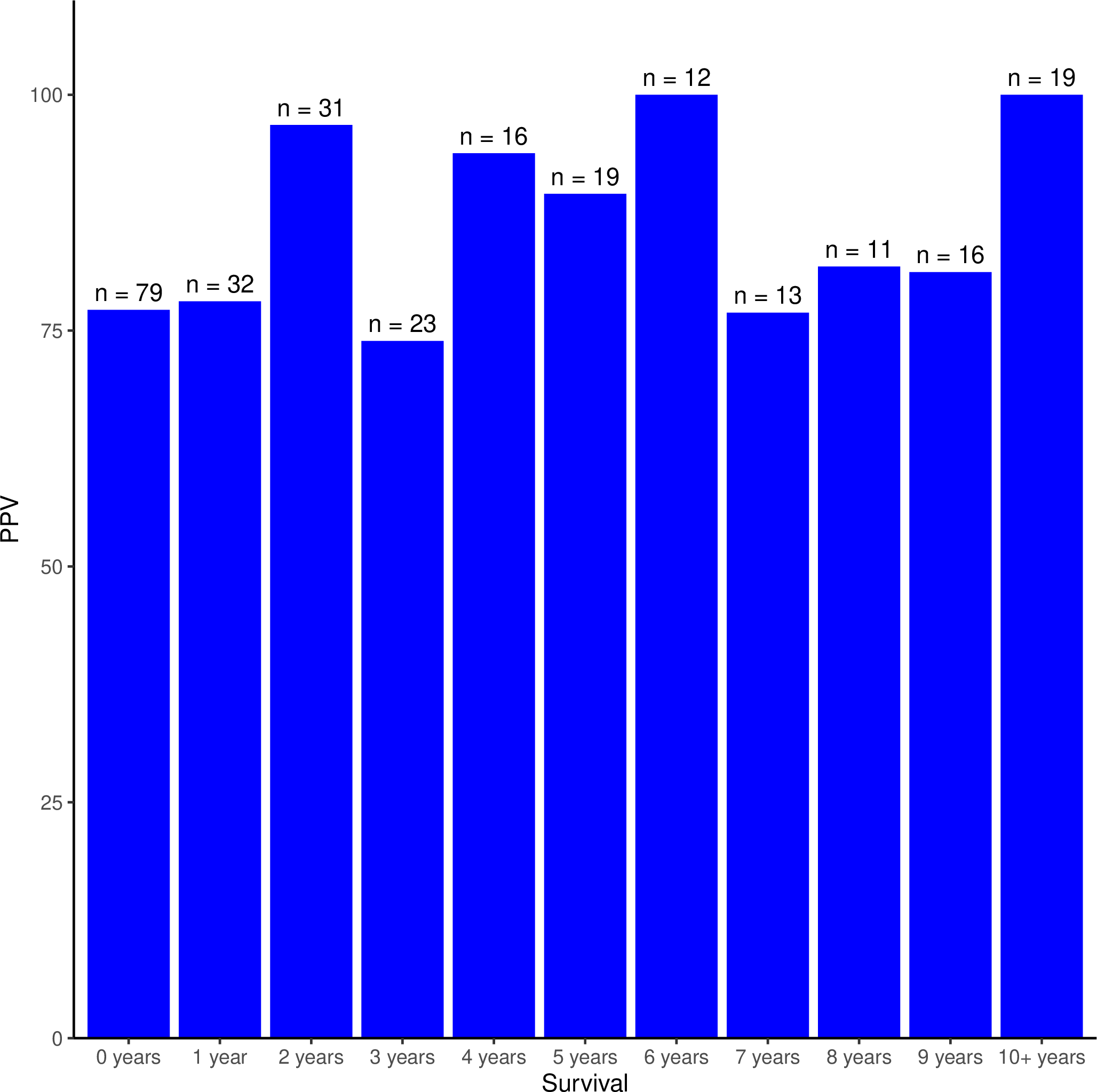

Supplement: S1 Fig — Bar chart showing the PPV of our broadest algorithm, stratified by patient survival time in years following the first seizure-related code. (TIF) [file pone.0329012.s001.tif]
