# Supplementary material for: Detecting poststroke epilepsy in nationwide administrative data: A validation study using Swedish registers
Source: PLoS One. 2025 Aug 12;20(8):e0329012. doi: 10.1371/journal.pone.0329012 (PMC12342257; doi:10.1371/journal.pone.0329012)
Supplement: S1 Table — (PDF) [file pone.0329012.s002.pdf]

S1 Table. Comparison of characteristics between individuals included in the study and those whose records were not ordered or received.

	Included n=321	No available medical records n=179	*P*
Age at first seizure-related diagnostic code, years (median, IQR)	78 (70-84)	80 (72-85)	.22
Time between stroke and first seizure-related diagnostic code, days (median, IQR)	326 (142-700)	280 (92-848)	.49
Sex	n (%)	n (%)	.08
Female	140 (44)	93 (52)	
Male	181 (56)	86 (48)	
Diagnostic code for epilepsy (G40)	245 (76)	132 (74)	.52
Dispensed ASM	258 (80)	144 (80)	1.00
Access to neurology care‡	106 (33)	63 (35)	.62

‡ Seizure-related diagnostic code registered at a neurology clinic or an ASM prescribed by a neurologist.
